# Optimizing Fluid Resuscitation Strategies: A Network Meta-analysis of Effectiveness and Safety for Hemorrhagic Shock Patients in Emergency Settings

**DOI:** 10.5811/westjem.47198

**Published:** 2025-11-26

**Authors:** Fan Maitri Aldian, Visuddho Visuddho, Michelle Vanessa Anggarkusuma, Jesphine Arbi Wijaya, Anthony Camilo Lim, Galen Chandrawira, Yan Efrata Sembiring, Bambang Pujo Semedi, Jeffrey Jeswant Dillon

**Affiliations:** * Universitas Airlangga, Faculty of Medicine, Surabaya, Indonesia; † Brawijaya University, Faculty of Medicine, Malang, Indonesia; ‡ Diponegoro University, Faculty of Medicine, Semarang, Indonesia; § Universitas Airlangga, Department of Thoracic Cardiac and Vascular Surgery, Surabaya, Indonesia; ¶ Dr Soetomo General Academic Hospital, Department of Anesthesiology and Intensive Care, Surabaya, Indonesia; || Dr Soetomo General Academic Hospital, Department of Thoracic Cardiac and Vascular Surgery, Surabaya, Indonesia; # Universitas Airlangga, Department of Anesthesiology and Intensive Care, Surabaya, Indonesia; ** Institut Jantung Negara, Department of Cardiology and Department of Cardiothoracic Surgery, Kuala Lumpur, Malaysia

## Abstract

**Introduction:**

Hemorrhagic shock is a life-threatening condition and remains a leading cause of death worldwide. Current European guidelines lack recommendations for one fluid type over another in the management of hemorrhagic shock. This study explores the effectiveness and safety of colloids and crystalloids in resuscitation of hemorrhagic shock patients.

**Methods:**

We conducted a systematic search in PubMed, Cochrane Cenral Register of Controlled Trials (CENTRAL), Scopus, Web of Science, ProQuest, and Cumulative Index to Nursing and Allied Health Literature (CINAHL) up to January 3, 2024. We performed data analyses using Rstudio v.4.4.1 in Frequentist network meta-analysis with DerSimonian-Laird random-effects model. Subgroup and network meta-regression analyses was also performed in Bayesian methods. We analyzed safety aspects using meta-proportions with generalized linear mixed models models.

**Results:**

A total of 3,693 patients from 23 randomized controlled trials were included in this study. Synthetic colloid demonstrated the lowest mortality rate (odds ratio 0.37, 95% CI, 0.15–0.93; *P-score* = .94) with the lowest fluid input requirement (mean difference −1.02; 95% CI, −1.62 to −0.41; *P-score* = .75). Subgroup and network meta-regression analysis revealed none of the covariates significantly influenced these two outcomes. Regarding safety aspects, isotonic crystalloid caused the most diverse adverse events, with acute respiratory distress syndrome (prop = 0.067) and overload syndrome (prop = 0.063) being the most common adverse events.

**Conclusion:**

This study provides robust evidence favoring the initial use of synthetic colloid in the management of patients with hemorrhagic shock.

## INTRODUCTION

Hemorrhagic shock is a life-threatening, medical emergency condition of intravascular volume depletion through blood loss, leading to inadequate perfusion of tissues and organs to oxygen.^1^ If left uncorrected, acidosis with persistent hypoxemia will eventually cause the loss of peripheral vasoconstriction, worsening hemodynamic compromise, and death. Hemorrhagic shock remains a leading cause of death worldwide, which is responsible for over 80% of deaths in the operating room and nearly 50% of deaths within 24 hours after injury.^2^

The current suggestion for hemorrhagic shock management is based on a timely, rapid, definitive source control of bleeding and blood loss replacement.^3^ Therefore, fluid resuscitation emerges as a cornerstone of effective medical management, highlighting its vital role in restoring intravascular volume, maintaining tissue perfusion, and preventing the onset of shock and organ failure, particularly in situations where blood is not available.^4^ The most frequently used types of fluid for resuscitation are crystalloid and colloid solutions.^5^ Crystalloid solutions that mimic the electrolyte composition of plasma, such as normal saline, Ringer lactate, bicarbonated Ringer solution, hypertonic saline, Ringer acetate, and Plasma-Lyte A, are associated with lower incidence of acute kidney injury, decreased need for renal-replacement therapy, and reduction in mortality.^6^

In comparison to colloids, however, resuscitation using crystalloids requires two to four times more fluid to replenish and sustain intravascular fluid volume, which dilutes plasma proteins, lowering colloid osmotic pressure, and quickly leaks into the interstitial space.^7^,^8^ On the other hand, colloids are solutions containing high molecular weight substances, which do not easily cross capillary membranes. Colloids, including dextran, mannitol 15%, hydroxyethyl starch, plasma, albumin, gelatin, and hypertonic colloid, increase survival rates in several studies.^5^,^9^ Colloid osmotic pressure in the plasma helps keep fluids inside the blood vessels, resulting in quicker and more effective resuscitation.^5^,^9^

Nevertheless, current European guideline for traumatic hemorrhagic shock noted that there is no accepted intervention regarding the superiority of one among the others.^10^ Although many studies have been conducted to compare types of fluid interventions, a quantitative analysis comparing all available interventions has never been conducted. Our goal in this study was to provide evidence and fill the knowledge gaps regarding the effectiveness and safety of each fluid resuscitation strategy in improving hemorrhagic shock-related patient outcomes.

## METHODS

We conducted this systematic review and network meta-analysis (NMA) in accordance with the Preferred Reporting Items for Systematic Reviews and Meta-Analyses (PRISMA) NMA Checklist of Items (Table S1).^11^ The study protocol has been registered on PROSPERO (Record No. CRD42024516480).^12^

### Search Strategies

We conducted a computerized systematic literature data search in PubMed, Scopus, CENTRAL, CINAHL, ProQuest, and Web of Science up to January 3, 2024. To ensure comprehensive coverage, we established a list of primary keywords that included “hemorrhagic shock,” “crystalloid,” “colloid,” and “resuscitation.” We then added several Medical Subject Headings and other accessible text terms to construct the database-specific search terms. The entire search terms for each database are provided in Table S2.

**Population Health Research Capsule**

What do we already know about this issue?

*Current fluid selection for hemorrhagic shock largely depends on availability, and existing guidelines lack firm recommendations.*

What was the research question?

*Which fluid type is most effective in reducing mortality and ensuring safety in hemorrhagic shock resuscitation?*

What was the major finding of the study?

*Synthetic colloids showed the lowest mortality (OR 0.37, 95% CI 0.15–0.93; P-score = .94) and reduced fluid input (MD −1.02; 95% CI −1.62 to −0.41).*

How does this improve population health?

*Provides evidence-based guidance for optimal fluid selection in hemorrhagic shock, improving survival and emergency care outcomes.*

### Study Selection, Eligibility Criteria, and Data Extraction

We used the Population, Intervention, Comparison, Outcome (PICO) framework^13^ (Table 1), which is specifically designed for systematic reviews to establish the eligibility criteria. The full inclusion and exclusion criteria of the studies are depicted in Table 2. A full explanation of study selection and data extraction can be accessed in Supplementary Material.

### Quality Assessment of Individual Studies

Risk of bias of each eligible study is evaluated by the Cochrane risk of bias 2 (RoB-2) tool.^14^ The RoB-2 plots were generated using the “robvis” software.^15^

### Statistical Analysis

We performed NMA using Frequentist^16^ methods with netmeta^17^ packages in Rstudio v4.4.1 (Posit, Boston, MA,).^18^ We conducted NMA using pooled mean difference (MD) and odds ratio (OR) based on the reported type. The DerSimonian-Laird random-effects model was used to accommodate unavoidable heterogeneity.^19^ A higher *P*-score indicates a better treatment for that outcome. Publication bias was assessed both qualitatively using an inverted funnel plot and quantitatively using the Egger regression test.^20^ Heterogeneity was examined with Cochran’s *Q* statistics.^21^ The inconsistency assessment was conducted using global and local approaches.^19^ The local inconsistency assessment is carried out using the Separating Indirect from Direct Evidence (SIDE) method.^22^ Global inconsistency was assessed using between designs *Q* statistics value.^23^ The Grading of Recommendations, Assessment, Development and Evaluation) to rate the quality of evidence (GRADE) methodology evaluates findings based on six domains: within-study bias based on the RoB-2 tool assessment; reporting bias; indirectness; imprecision; heterogeneity; and incoherence.^24^ Frequentist framework is preferred for the primary NMA to ensure accessibility and interpretability for a broad readership, as it provides confidence intervals and *P*-values that are widely recognized in clinical research. We applied Bayesian models for subgroup and meta-regression analyses because of their greater flexibility in exploring effect modification and complex relationships. Both approaches yielded consistent conclusions, supporting the robustness of our findings.

### Meta-proportions for Analyzing Safety Outcomes

We performed meta-proportion analysis using generalized linear mixed models (GLMM) methods.^25^ Meta-analyses of proportions focuses on estimating the overall (median or population-averaged) proportion, regardless of the transformation used. In this sense, they differ from meta-analyses of treatment comparisons, which may aim at estimating different relative effects (eg, OR, risk ratio or risk difference), depending on how event rates are transformed.^26^,^27^

In a network meta-analysis, the P-score indicates the relative ranking of each treatment, ranging from 0 to 1, with higher scores reflecting more favorable treatments.

## RESULTS

### Overview of Study Selection Process

**Figure 1:**
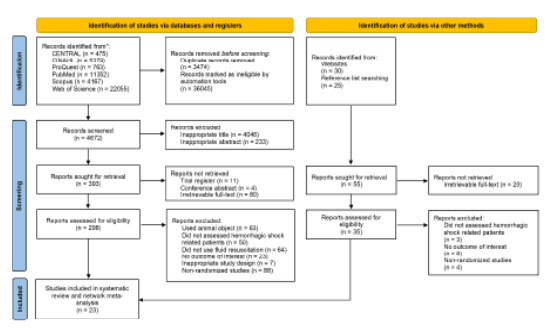
PRISMA* flow diagram of the study selection process. *PRISMA, **Preferred Reporting Items for Systematic Reviews and Meta-Analyses.** *CENTRAL*, Cochrane Central Register of Controlled Trials; *CINAHL*, Cumulative Index to Nursing and Allied Health Literature.

A PRISMA flowchart of the entire study selection process is depicted in Figure 1. The initial search of the six databases yielded 44,191 hits. Among these, we removed 3,474 duplicates; and 36,045 records were found to be ineligible by the automated tool. Of the remaining 4,672 studies, 4,046 and 233, respectively, were excluded based on their title and abstracts. Neither did we retrieve 11 trial registers, four conference abstracts, and 80 articles with no available full-text. We conducted a detailed examination of the full text of the remaining 298 studies, which resulted in the exclusion of 236 studies due to the following: inappropriate population with animals as study subjects (n = 63) and not assessing hemorrhagic shock-related patients (n = 50); not intervening with fluid resuscitation (n = 64); not reporting the outcome of interest (n = 23); inappropriate study design (n = 7); and non-randomized studies (n = 88). In addition to database searching, we identified 55 additional records from websites and reference lists searches, of which 20 reports were not retrieved. After assessing the eligibility of the remaining reports, we excluded 15. Finally, a total of 23 randomized controlled trials (RCT)^28^–^50^ were included in this systematic review and NMA.

### Characteristics and Baseline Outcomes of Included Studies

The characteristics of the included studies are presented in Table 3. A total of 3,693 patients were included across 23 RCTs, with participants ranging from 31.3–48.25 years of age. Of these patients, 1,048 were female, representing 28.37% of the study population. Study sample sizes varied from 23 to 626 patients. The included studies were geographically diverse, spanning four continents, with two studies from Africa, 14 from America, five from Asia, and two from Europe. The included studies assessed two types of patient characteristics, including hemorrhagic shock patients (n = 12) and trauma hemorrhagic shock patients (n = 11). The average adherence rate of the included studies was 89.69%. Most of the studies performed intention-to-treat analysis (n = 21), while the remaining used per-protocol analysis (n = 2). Patient baseline data from each study can be seen in Table 4. The data include information related to the shock hemorrhagic condition: Injury Severity Score (ISS); revised trauma score; Glasgow Coma Scale (GCS), time to point of injury to arrival on scene; time from point of injury to the emergency department; patient’s hemodynamics (pH, hemoglobin levels, lactate levels, heart rate, systolic blood pressure, mean arterial pressure, central venous pressure, osmolarity, and hematocrit); and electrolyte balance (sodium, potassium, chloride, and bicarbonate levels).

### Quality Assessment of Included Studies

The results of the domain-specific quality assessment are presented in Figure S1, while the ROB evaluation for each study is summarized in Figure S2. Based on Cochrane’s ROB-2 tool, six and 17 studies demonstrated low and moderate risk of bias in all domains, respectively.

### Network Meta-Analysis and Rank Probability

The established network plots for fluid resuscitation are displayed in Figure S3. The detailed results of each fluid resuscitation are displayed in the form of pairwise meta-analysis, which can be seen in Figure S4–S5. Figure 2.1A shows the results of frequentist NMA for mortality rate outcome. The forest plot revealed that patients with synthetic colloid resuscitation (OR 0.37, 95% CI, 0.15–0.93, P = .94 had the lowest mortality rate and also required the least resuscitation volume (MD −1.02, 95% CI, −1.62 to −0.41; P = .75) compared to the others. Meanwhile, use of natural colloid also proved to result in significantly lower mortality rates (OR 0.70; 95% CI, 0.55–0.88, P = .63). However, Figure 2.1B, shows that those who received natural colloid (OR 0.04, 95% CI, −1.06 to 1.13, P = .17) needed more volume of fluid resuscitation compared to those receiving combination (OR −0.96, 95% CI, −1.67 to −0.24, P = .71) or hypertonic crystalloid resuscitation (OR −1.00; 95% CI, −2.03 to 0.03, P = .73).

Funnel plots results show no publication bias in all outcomes, including mortality rate and total fluid input (refer to Figure 2.2AB). The results of the frequentist NMA are also presented in the form of a league table in Table S3, while summary of the P-values, which display the probabilities for each treatment to the outcomes, are depicted in Table 5. The results of the funnel plots agreed with the results of the Egger test, boh indicating no publication bias within the mortality rate (P = .99) and total fluid input (P = .99) outcomes.

Negligible heterogeneity level was found in mortality rate NMA (refer to Table S4). However, moderate to high heterogeneity was discovered between combination and isotonic crystalloid (I^2^ = 86.4%), between synthetic colloid and isotonic crystalloid (I^2^ = 53.3%). The SIDE methods revealed inconsistency was not present in all comparisons (Table S4). This result agrees with the global inconsistency result, which also found no significant inconsistency within mortality rate (Q = 5.83, P = .05) and total fluid input (Q = 1.12, P = .57) (Figure S3–S4). The direct and indirect evidence plot of each network meta-analysis also presented in Figure S8 and Figure 9, demonstrate that the evidence of some comparisons are limited, whether it was obtained by either direct or indirect, while others are evidenced by both direct and indirect.

### Adverse Effects

The results of meta-proportions on adverse effects from every fluid resuscitation group are summarized in Table 6. Among all the fluid groups, isotonic crystalloid was associated with the most diverse adverse events, with acute respiratory distress syndrome (proportion = 0.067) and overload syndrome (proportion = 0.063) being the most common adverse events. Both natural and synthetic colloids also led to various side effects, with pneumonia (prop = 0.431) and acute respiratory distress syndrome (proportion = 0.103) being the most prevalent side effects, respectively. Lastly, combination therapy showed overload syndrome (proportion = 0.059) as the most prevalent adverse effect.

### Subgroup and Network Meta-Regression Analysis

Table S8 contains the results of the subgroup and network meta-regression analysis for mortality rate and total fluid input outcomes. The subgroup analysis revealed that no statistical difference was found in 1) continent, 2) patient characteristics, 3) type of analysis, and 4) risk of bias results between two outcomes. Subsequently, network meta-regression analysis also found that none of the covariates significantly influenced these two outcomes.

### Quality of Evidence

The within-study bias assessment revealed several concerns (Figure S10, S11). The indirectness in the network assessment is depicted in Figure S12, S13. Furthermore, the full GRADE report assessment identified significant issues related to imprecision, within-study bias, and heterogeneity, which can lead to a decrease in the confidence rating level (Table 7).

## DISCUSSION

Hemorrhagic shock is a critical condition in trauma and emergency settings, while crystalloids playing a significant role in volume resuscitation.^1^ In this study we compared the effectiveness of various resuscitation fluids, finding that synthetic colloids led to greater mortality reduction than isotonic crystalloids. Furthermore, synthetic colloids, hypertonic crystalloids, and colloid-crystalloid combinations significantly reduced overall fluid input compared to isotonic crystalloids. These results are consistent with the pathophysiology of hemorrhagic shock, supporting the use of appropriate fluids in clinical settings.

During hemorrhagic shock, the loss of circulating blood volume reduces oxygen supply to tissues, triggering compensatory mechanisms such as vasoconstriction, increased heart rate, and activation of the renin-angiotensin-aldosterone system.^51^ While these responses help maintain perfusion to vital organs, tissue hypoxia eventually leads to anaerobic metabolism, lactic acidosis, and organ failure. Prompt volume restoration is essential to reverse these processes.^52^

### Mortality

**Figure 2:**
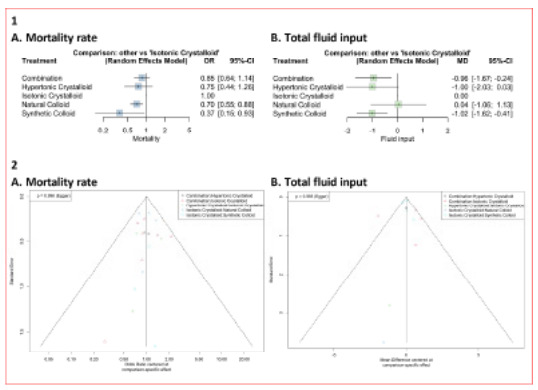
Forest plot of relative effect (1A and 1B) and funnel plot (2A and 2B) from network meta-analysis using a frequentist random-effects model for mortality rate (A) and total fluid input (B). The vertical line indicates the line of no effect. Values to the left of the line favor the compared interventions, while values to the right favor the reference interventions. *MD*, mean difference; *OR*, odds ratio.

Our study found that synthetic colloids significantly reduced mortality compared to isotonic crystalloids (Figure 2A). Colloids are known for their superior volume-expansion properties, requiring smaller volumes to achieve the same hemodynamic effect as crystalloids.^9^ However, while they are effective in trauma settings by maintaining adequate intravascular volume and tissue perfusion, colloids can increase the risk of renal complications.^53^ Nevertheless, newer generation starch-based synthetic colloids can stabilize hemodynamics with fewer complications in trauma-induced hemorrhagic shock.

Synthetic colloids have been shown to stabilize key hemodynamic parameters, such as mean arterial pressure (MAP) and central venous pressure (CVP), more effectively than crystalloids.^54^ Early stabilization of MAP and CVP correlates with improved survival and a lower incidence of multiorgan failure. By reducing the volume needed to restore circulation, synthetic colloids also minimize tissue edema and prevent dilutional coagulopathy.^55^,^56^ Additionally, the reduced fluid input helps maintain serum bicarbonate levels, stabilizing pH and reducing lactate accumulation, which is a key indicator of anaerobic metabolism.^57^ Studies suggest that rapid restoration of hemodynamics with these fluids limits the extent of metabolic acidosis.^58^

### Fluid Input

Our analysis also revealed that synthetic colloids, hypertonic crystalloids, and colloid-crystalloid combinations have more reduction on total fluid input compared to isotonic crystalloids (Figure 2B). The colloid-crystalloid combinations demonstrate the synergism between colloids, which have a longer lasting volume-expanding effect and crystalloids, which rapidly distribute into both the intravascular and interstitial spaces.^59^ Hypertonic crystalloids, such as 3% sodium chloride, require smaller volumes due to their osmotic effect, which pulls fluid into the intravascular space from the interstitial and intracellular compartments. The use of hypertonic crystalloids reduce edema formation, which is beneficial in the resuscitation,^60^ as opposed to the isotonic crystalloid administration, which results in approximately 75% of the fluid being distributed in the interstitial space.^61^

Another reason that fluids like synthetic colloids, hypertonic crystalloids, and colloid-crystalloid combinations reduce fluid input may be a result of their positive impact on sodium and potassium balance. The effectiveness in electrolyte control reduces the need for excess fluid.^10^ Their role in maintaining a stable electrolyte profile prevents the dilutional hyponatremia and hyperkalemia, which increase the need of fluid input that occurs with isotonic solutions.^58^ Studies showed a correlation between elevated serum potassium levels and early mortality, as well as its association with prolonged resuscitation efforts.^62^

### Meta-Regression

Meta-regression analysis indicates that the use of these fluid resuscitation is both applicable and safe for patients of all genders and ages who suffer from hemorrhagic shock. These results align with previous studies, which found that despite differences in the fluid composition, there was no significant difference in resuscitation outcomes.^63^ Further analysis of any others shock-related outcomes, such as ISS, GCS, prehospital time, blood pressure, pH, hemoglobin levels, and lactate levels also revealed that the effectiveness of fluid resuscitation applied to hemorrhagic shock patients with a variety of diverse characteristics.

### Adverse Effect

As the significant portion of isotonic crystalloid fluids distribute into the interstitial space, large volumes are often required to achieve adequate resuscitation.^64^ The need to administer large volumes of isotonoic crystalloid fluids in hemorrhagic shock can lead to hemodilution. Hemodilution of blood components impairs hemostasis due to coagulation factor depletion.^65^ Hemodilution is associated with low oxygen-carrying capacity of blood, increasing the risk of acute kidney injury.^66^ The hemodiluted patients were more susceptible to bloodstream infection due to impairment of immune response, although this is still more related to the cause of hemorrhagic shock and aseptic technique.^67^ This aligns with European Society of Intensive Care Medicine guidelines, which conditionally recommend using balanced crystalloids instead of isotonic saline for patients with sepsis or kidney injury.^68^ Our findings demonstrated that natural colloids increase blood viscosity, which can elevate the risk of thrombosis. However, the effects of colloids on blood coagulation vary depending on the type of colloid used. The synthetic colloid hydroxyethyl starch has been linked to increased pulmonary capillary permeability and greater pulmonary leak index. This explains the high risk of acute respiratory distress syndrome in the synthethic colloid groups.^69^

## LIMITATIONS

To the best of our knowledge, this study is the first systematic review and network meta-analysis to analyze both the effectiveness and safety of any fluid resuscitation in hemorrhagic shock-related patients. Previously, the National Association of Emergency Medical Services Physicians published a statement paper that provides recommendations and position statements based on a broad synthesis of the available literature.^70^ In contrast, our study employed a rigorous network meta-analysis that included only randomized controlled trials, enabling both direct and indirect comparisons across all major fluid types. This approach addresses a specific evidence gap by offering a comprehensive, head-to-head evaluation of fluid resuscitation strategies. We believe that our findings not only complement existing position statements but also contribute novel, quantitative comparative evidence that may inform future clinical guideline development in trauma resuscitation. Furthermore, the study also covered a wide range of areas and included a sufficient number of samples for all analyses, which will enhance the study’s findings.

Despite our attempts to deliver the best possible quality of study, we acknowledge areas for improvement, including some concerns that we found in imprecision, within-study bias, and heterogeneity evaluation, which resulted in a downgrade in the confidence rating. We also included seven studies that are over 20 years old; we conducted a network meta-regression analysis to assess whether the year of publication had a significant influence on the study outcomes. The results of this analysis indicated no significant effect of publication year on the network estimates, thus mitigating concerns about the potential impact of older studies on our overall findings.

We also acknowledge the study by Andrews et al, which demonstrated that aggressive fluid resuscitation in adults with sepsis and hypotension in low-resource settings was associated with increased harm. However, this population differs substantially from patients with hemorrhagic shock, where the underlying pathophysiology, resuscitation goals, and standard of care are distinct. While the findings raise important considerations about fluid overuse in resource-limited environments, their applicability to hemorrhagic shock remains limited.^71^ Furthermore, Andrews’ study primarily focused on crystalloid-based strategies, whereas blood products remain the cornerstone of resuscitation in hemorrhagic shock. The precise role of crystalloids when used in conjunction with blood products is not fully established, and addressing this question was beyond the scope of the present analysis.

## CONCLUSION

Our study supports the use of synthetic colloids, including hydroxyethyl starch and gelatin, as the first choice in treating patients with hemorrhagic shock-related disease due to associated decreases in mortality and adverse events. Considering the accessibility of colloids, the use of a combination of colloids and crystalloids may be an option. It would be beneficial for future studies to analyze the long-term outcomes of fluid resuscitation, such as the need for renal replacement therapy, as well as evaluate the efficacy of whole blood in hemorrhagic shock resuscitation. It is imperative to consider the cost implications, accessibility, and clinical suitability inherent to each fluid type.

## Supplementary Information



## Figures and Tables

**Table 1:** Characteristics of studies, using the PICO* framework to explore the effectiveness and safety of colloids and crystalloids in resuscitation of hemorrhagic shock patients.

Components of PICO	Definition
**P**opulation	Adult patients with hemorrhagic shock
**I**ntervention	Isotonic Crystalloid (RL, Plasma-Lyte A, BRS) Hypertonic Crystalloid (HS) Natural Colloid (plasma, albumin) Synthetic Colloid (HES, gelatin) Combination (HH, HSD)
**C**omparison	Isotonic crystalloid: Normal saline
**O**utcome	Primary outcomes Mortality rate and total fluid input Secondary outcomes Any adverse effects due to fluid resuscitation

* *PICO*, Population, Intervention, Comparison, and Outcome.

*RL*, Ringer lactate; *BRS*, bicarbonated Ringer solution; *HS*, hypertonic saline; *HES*, hydroxyethyl starch; *HH*, hypertonic saline + hydroxyethyl starch; *HSD*, hypertonic saline dextran.

**Table 2:** Summary of *P*-values for mortality rate and total fluid input network meta-analysis.

A. Mortality rate	
Treatment	*P*-score
Synthetic colloid	**.94**
Natural colloid	.63
Hypertonic crystalloid	.52
Combination	.34
Isotonic Crystalloid	.07
B. Total fluid input	
Treatment	*P*-score
Synthetic colloid	**.75**
Hypertonic crystalloid	.73
Combination	.71
Natural colloid	.17
Isotonic cystalloid	.14

Higher *P*-value indicates a better treatment for that outcome.

**Table 3:** Summary of meta-proportions on adverse effects from fluid resuscitation.

Adverse effect	Isotonic crystalloid			Natural colloid			Synthetic colloid			Combination		
	k	Proportion	95% CI	k	Proportion	95% CI	k	Proportion	95% CI	k	Proportion	95% CI
Acute kidney injury	2	0.012	0.000; 0.966	-	-	-	-	-	-	1	0.009	0.000; 0.054
Acute respiratory distress syndrome	6	0.067	0.015; 0.260	1	0.043	0.023; 0.081	2	0.103	0.002; 0.867	-	-	-
Bloodstream infection	2	0.017	0.000; 0.913	1	0.014	0.003; 0.053	-	-	-	-	-	-
Coagulopathy	3	0.046	0.013; 0.147	-	-	-	-	-	-	1	0.009	0.001; 0.034
Overload syndrome	1	0.063	0.026; 0.126	-	-	-	-	-	-	1	0.059	0.022; 0.125
Pneumonia	3	0.015	0.002; 0.112	1	0.431	0.317; 0.553	-	-	-	2	0.018	0.000; 0.918
