# Supplementary material for: Optimizing Fluid Resuscitation Strategies: A Network Meta-analysis of Effectiveness and Safety for Hemorrhagic Shock Patients in Emergency Settings
Source: West J Emerg Med. 2025 Nov 26;26(6):1795–803. doi: 10.5811/westjem.47198 (PMC12698173; doi:10.5811/westjem.47198)
Supplement: Supplementary file 1 [file wjem-26-1795-s001.pdf]

SUPPLEMENTARY DATA

DATA AVAILABILITY

METHODS

Selection of Studies

Search results from each database were collected and managed using Google Sheets (https://docs.google.com/spreadsheets/) (Google LLC, Mountain View, CA, USA). After deduplication, the remaining articles were selected based on title and abstract. All articles included in the next screening step were retrieved. After that, we find studies with available and published full-texts and thoroughly assessed them according to the pre-specified eligibility criteria. The reasons for excluding each article from each screening step were reported as appropriate in the spreadsheet. The literature searches and overall study selection process were completed by three investigators (FMA, JAW, MVA). Any disagreements were reconciled through a group discussion.

Eligibility Criteria

To be included in this study, studies had to meet the following criteria: (1) consisted of hemorrhagic shock-related patients; (2) intervention using isotonic crystalloid (ringer's lactate, plasma-lyte A, bicarbonated ringer's solution) or hypertonic crystalloid (hypertonic saline) or natural colloid (plasma, albumin) or synthetic colloid (hydroxyethyl starch, gelatin) or combination (hypertonic saline dextran, hypertonic saline + hydroxyethyl starch); (3) implemented normal saline for the control group; (4) the outcome measured are efficacy (mortality, fluid requirement) and safety (any adverse effects) from fluid resuscitation; (5) employed RCTs. Studies were excluded if: (1) the title or abstract was irrelevant; (2) irretrievable full-texts; (3) were review article, case report, case series, or conference abstract; (4) were animal or in vitro study.

Data Extraction

A team of three investigators, consisting of FMA, JAW, and MVA, meticulously extracted data from each included study using a pre-specified checklist developed and tabulated within the spreadsheet by FMA. After that, JAW and MVA checked the collected data for their eligibility, and any disagreements were promptly resolved. The data extracted include the name of the first author and year of publication, title of article,

DOI, group of therapy (colloid, crystalloid, or combination), type of therapy, regimen of therapy, trial identifier, study location, patient characteristics, diagnostic criteria, mean age of patients, sample size, participants dropped-out in intervention group, adherence rate, percentage of female, and type of analysis (intention to treat [ITT] or per-protocol [PP]), type of injury, injury mechanism, injury score ([abbreviated injury scale] AIS-head & neck, AIS-traumatic brain injury, AIS-chest, AIS-abdomen & pelvis, AIS-extremities, ISS [injury severity score], RTS [revised trauma score], and TRISS [trauma and injury severity score]), shock index (SI), degree of shock, glasgow coma scale (GCS), prehospital time (time from point of injury (POI) to arrival on scene (AOS), time from POI to emergency department (ED), time from AOS to ED), pH, hemoglobin (HGB) levels, international normalized ratio (INR), blood lactate, transfussion in 24 hours, packed red blood cell (pRBC) in 24 hours, total crystalloid in 24 hours, input fluid, output fluid, fluid balance, heart rate, body temperature, base excess, systolic blood pressure (SBP), diastolic blood pressure (DBP), mean arterial pressure (MAP), mean pulmonary artery pressure (mPAP), central venous pressure (CVP), pulmonary vascular resistance (PVR), stroke volume index (SVI), oxygen delivery (DO2), oxygen consumption (VO2), Na, K, Cl, PCO2, HCO3, osmolarity, hematocrit (HCT), partial thromboplastin time (PTT), platelet (PLT), fibrinogen, coagulation factor II, V, VII, VIII, IX, and XI, total hospital stays, mortality rate, time to achieve resuscitation, pulmonary capillary wedge pressure (PCWP), systemic vascular resistance (SVR), total fluid input, total participants who need transfusion, and any adverse effects of fluid intervention. Only data from studies with sufficient coverage were included and presented in a tabular format with qualitative characteristics and outcomes.

Quality Assessment of Individual Studies

Two assessors (GC and ACL) independently conducted a methodological quality assessment to evaluate the risk of bias of each eligible study using the Cochrane Collaboration's Risk of Bias 2 (RoB 2) tool. Disagreements of judgements were resolved by a third assessor (FMA). The RoB 2 is a revised tool consisting of five bias domains explicitly designed to consider the risk of bias of randomized trials arising from: (1) the randomization process; (2) deviations from intended interventions; (3) missing outcome data; (4) the measurement of the outcome; and (5) the selection of the reported result. The risk of bias on each domain was rated as low risk, high risk, or some concerns (unclear)

to the algorithms that incorporated several domain-specific signaling questions. Judgment levels from all domains were later deduced as an overall risk of bias for each study. A study is considered low risk of bias if all domains show low risk. If at least one domain was rated as unclear, studies were judged as having some concerns. Studies were judged to be at high risk of bias if at least one domain presents a high risk or there were some concerns in multiple domains that could significantly lower the confidence in the study results.

Statistical Analysis

Total fluid input outcomes were reported in Mean Difference (MD). As for dichotomous outcomes, OR was used to report the mortality rate. NMA are presented in the Frequentist method, including forest plot, funnel plot, netsplit plot, effect estimate table, net table, decomp design, contribution matrix, and direct & indirect evidence plot. The pooled effect size and heterogeneity assessment results from each comparison are obtained from the pairwise forest plot. In contrast, the frequentist forest plot compares the effect size of each treatment with isotonic crystalloid as the reference treatment. Additionally, the results of the network meta-analyses are presented in the form of a league table, which compares the pooled effect size results of all pairwise comparisons in the network meta-analyses.

Subgroup and Network Meta-Regression Analysis

The subgroup and network meta-regression analyses were performed in Bayesian network meta-analysis. MCMC simulation for the subgroup and network meta-regression analysis, we produced four chains of 20,000 samples (20,000 Markov Chain Monte Carlo simulations, keeping every 1st iteration). The probability that intervention was numerically better than comparison, estimated from the proportion of Markov Chain Monte Carlo cycles, was calculated. Convergence was assessed by visual inspection of trace plots and confirmed using the Gelman–Rubin diagnostic, with all values <1.05 indicating satisfactory convergence. Diagnostic checks confirmed model stability, and no evidence of lack of convergence was observed. The probability that an intervention was superior to a comparator was estimated from the proportion of MCMC iterations favoring that intervention. Statistical significance was defined as 95% credible interval (CrI) not cross the line of no effect (OR = 1, and MD = 0). The lower and upper bounds of the credible interval were set to 2.5% and 97.5% quantiles derived from the Bayesian

inference. Credible interval (CrI) captured the uncertainty of parameters based on the posterior distribution, whereas confidence interval captured the uncertainty of parameters based on repeated sampling. We performed subgroup analyses in four variables in each outcome, including (1) continent, (2) patient characteristics, (3) type of analysis, and (4) risk of bias result. To evaluate the difference in effect size between certain covariates and the pooled outcome, we performed network meta-regression on several covariates, including: (1) Publication year, (2) sample size, (3) mean age, (4) adherence rate, (5) female percentage, (6) ISS, (7) GCS, (8) baseline hemoglobin levels, (9) baseline pH, (10) time from POI to ED, (11) baseline blood lactate levels, and (12) baseline SBP.

Heterogeneity and Inconsistency Assessment

Heterogeneity assessment has two components: within-design heterogeneity and inconsistency between designs. The SIDE assessment is performed using nettable() command in netmeta package. The results of the inconsistency assessment for each pairwise are then displayed in the form of a netsplit forest plot, where a p-value <0.05 is used to indicate statistically significant inconsistency throughout the analysis. The global inconsistency was calculated using decomp.design() command in Rstudio to obtain the between designs Q statistics value. A p-value <0.05 is used to indicate a significant inconsistency in the outcome.

GRADE Report

To assess the confidence of the results of each network meta-analysis, each finding was analyzed using CINeMA, an adaptation of the GRADE Report approach. If no concerns exist in any of these domains, the confidence level would be rated as "High". If there are some concerns in 1 or 2 domains, the confidence level would be rated as "Moderate". However, if there are major concerns in one domain or multiple concerns across three domains, the trust level would decrease to "Low". In cases where there are major concerns and some concerns across multiple domains, the trust level would be rated as "Very low".

Table S1. PRISMA NMA Checklist of Items to Include When Reporting A Systematic Review Involving a Network Meta-analysis

**Section/Topic**	**Item #**	**Checklist Item**	**Reported on Page #**
**TITLE**			
Title	1	Identify the report as a systematic review *incorporating a network meta-analysis (or related form of meta-analysis).*	**1**
**ABSTRACT**			**1**
Structured summary	2	Provide a structured summary including, as applicable:<br>**Background:** main objectives<br>**Methods:** data sources; study eligibility criteria, participants, and interventions; study appraisal; and *synthesis methods, such as network meta-analysis.*<br>**Results:** number of studies and participants identified; summary estimates with corresponding confidence/credible intervals; *treatment rankings may also be discussed. Authors may choose to summarize pairwise comparisons against a chosen treatment included in their analyses for brevity.*<br>**Discussion/Conclusions:** limitations; conclusions and implications of findings.<br>**Other:** primary source of funding; systematic review registration number with registry name.	
**INTRODUCTION**			
Rationale	3	Describe the rationale for the review in the context of what is already known, *including mention of why a network meta-analysis has been conducted.*	**2**

Objectives	4	Provide an explicit statement of questions being addressed, with reference to participants, interventions, comparisons, outcomes, and study design (PICOS).	**2-3**
**METHODS**			
Protocol and registration	5	Indicate whether a review protocol exists and if and where it can be accessed (e.g., Web address); and, if available, provide registration information, including registration number.	**3**
Eligibility criteria	6	Specify study characteristics (e.g., PICOS, length of follow-up) and report characteristics (e.g., years considered, language, publication status) used as criteria for eligibility, giving rationale. *Clearly describe eligible treatments included in the treatment network, and note whether any have been clustered or merged into the same node (with justification).*	**3, Table S3, Table S4**
Information sources	7	Describe all information sources (e.g., databases with dates of coverage, contact with study authors to identify additional studies) in the search and date last searched.	**3**
Search	8	Present full electronic search strategy for at least one database, including any limits used, such that it could be repeated.	**3**
Study selection	9	State the process for selecting studies (i.e., screening, eligibility, included in systematic review, and, if applicable, included in the meta-analysis).	**4**
Data collection process	10	Describe method of data extraction from reports (e.g., piloted forms, independently, in duplicate) and any processes for obtaining and confirming data from investigators.	**4, Supplemental methods**

Data items	11	List and define all variables for which data were sought (e.g., PICOS, funding sources) and any assumptions and simplifications made.	**Table S3, 3-4**
**Geometry of the network**	**S1**	Describe methods used to explore the geometry of the treatment network under study and potential biases related to it. This should include how the evidence base has been graphically summarized for presentation, and what characteristics were compiled and used to describe the evidence base to readers.	**4**
Risk of bias within individual studies	12	Describe methods used for assessing risk of bias of individual studies (including specification of whether this was done at the study or outcome level), and how this information is to be used in any data synthesis.	**4**
Summary measures	13	State the principal summary measures (e.g., risk ratio, difference in means). *Also describe the use of additional summary measures assessed, such as treatment rankings and surface under the cumulative ranking curve (SUCRA) values, as well as modified approaches used to present summary findings from meta-analyses.*	**4**
Planned methods of analysis	14	Describe the methods of handling data and combining results of studies for each network meta-analysis. This should include, but not be limited to:<br>*Handling of multi-arm trials;*<br>*Selection of variance structure;*<br>*Selection of prior distributions in Bayesian analyses; and*<br>*Assessment of model fit.*	**4**
**Assessment of Inconsistency**	**S2**	Describe the statistical methods used to evaluate the agreement of direct and indirect evidence in the treatment network(s) studied.	**4**

		Describe efforts taken to address its presence when found.	
Risk of bias across studies	15	Specify any assessment of risk of bias that may affect the cumulative evidence (e.g., publication bias, selective reporting within studies).	**4**
Additional analyses	16	Describe methods of additional analyses if done, indicating which were pre-specified. This may include, but not be limited to, the following:<br>Sensitivity or subgroup analyses;<br>Meta-regression analyses;<br>*Alternative formulations of the treatment network; and*<br>*Use of alternative prior distributions for Bayesian analyses (if applicable).*	**4, Supplemental Methods**
**RESULTS†**			
Study selection	17	Give numbers of studies screened, assessed for eligibility, and included in the review, with reasons for exclusions at each stage, ideally with a flow diagram.	**5, Figure 1**
**Presentation of network structure**	**S3**	Provide a network graph of the included studies to enable visualization of the geometry of the treatment network.	**7, Figure 2**
**Summary of network geometry**	**S4**	Provide a brief overview of characteristics of the treatment network. This may include commentary on the abundance of trials and randomized patients for the different interventions and pairwise comparisons in the	**7-8**

		network, gaps of evidence in the treatment network, and potential biases reflected by the network structure.	
Study characteristics	18	For each study, present characteristics for which data were extracted (e.g., study size, PICOS, follow-up period) and provide the citations.	**6, Table S5, Table S6**
Risk of bias within studies	19	Present data on risk of bias of each study and, if available, any outcome level assessment.	**6-7, Figure S1, Figure S2**
Results of individual studies	20	For all outcomes considered (benefits or harms), present, for each study: 1) simple summary data for each intervention group, and 2) effect estimates and confidence intervals. *Modified approaches may be needed to deal with information from larger networks.*	**7-8, Figure S3-S4**
Synthesis of results	21	Present results of each meta-analysis done, including confidence/credible intervals. *In larger networks, authors may focus on comparisons versus a particular comparator (e.g. placebo or standard care), with full findings presented in an appendix. League tables and forest plots may be considered to summarize pairwise comparisons.* If additional summary measures were explored (such as treatment rankings), these should also be presented.	**7-9, Figure 3, Table S8, Table 1, Figure S5-S8**
**Exploration for inconsistency**	**S5**	Describe results from investigations of inconsistency. This may include such information as measures of model fit to compare consistency and inconsistency models, *P* values from statistical tests, or summary of inconsistency estimates from different parts of the treatment network.	**8-9, Figure S5-S6, Table S8**

Risk of bias across studies	22	Present results of any assessment of risk of bias across studies for the evidence base being studied.	**10, Figure S11-S12**
Results of additional analyses	23	Give results of additional analyses, if done (e.g., sensitivity or subgroup analyses, meta-regression analyses, *alternative network geometries studied, alternative choice of prior distributions for Bayesian analyses*, and so forth).	**8-12, Table 2, Table S9**
**DISCUSSION**			
Summary of evidence	24	Summarize the main findings, including the strength of evidence for each main outcome; consider their relevance to key groups (e.g., healthcare providers, users, and policy-makers).	**13-15**
Limitations	25	Discuss limitations at study and outcome level (e.g., risk of bias), and at review level (e.g., incomplete retrieval of identified research, reporting bias). *Comment on the validity of the assumptions, such as transitivity and consistency. Comment on any concerns regarding network geometry (e.g., avoidance of certain comparisons).*	**16**
Conclusions	26	Provide a general interpretation of the results in the context of other evidence, and implications for future research.	**16**
**FUNDING**			
Funding	27	Describe sources of funding for the systematic review and other support (e.g., supply of data); role of funders for the systematic review. This should also include information regarding whether funding has been received from manufacturers of treatments in the network	**N.A**

	and/or whether some of the authors are content experts with professional conflicts of interest that could affect use of treatments in the network.	

Table S2. Search Strategies

Database		Search Terms
Web of Science	#1	ALL=(Shock, Hemorrhagic)
	#2	ALL=(shock)
	#3	ALL=("circulatory failure" OR "circulatory collapse" OR "hypovolemic shock")
	#4	#2 OR #3
	#5	ALL=("trauma*" OR "injur*" OR "wound*")
	#6	#4 AND #5
	#7	#1 OR #6
	#8	(ALL=(Ringer's Lactate)) OR ALL=("Hartmanns Solution" OR "Lactate* Ringer*")
	#9	ALL=(Ringer's Acetate)
	#10	ALL=(Saline Solution)
	#11	ALL=(Albumins)
	#12	(ALL=(Hydroxyethyl Starch)) OR ALL=("Hydroxyethylated Starches" OR "2-Hydroxyethyl Starches" OR "Hemohes" OR "Elohes" OR "Hespan" OR "Hetastarch" OR "Pentafraction" OR "Pentaspan" OR "Pentastarch" OR "Plasmasteril" OR "HAES-steril")
	#13	(ALL=(Dextrans))
	#14	ALL=("Dextran" OR "Hemodex" OR "Hyskon" OR "Infukoll" OR "macrodex" OR "polyglucin" OR "Promit" OR "Rheoisodex" OR "Rondex" OR "Saviosol")
	#15	ALL=(Gelatin)
	#16	ALL=(Crystalloid Solutions)
	#17	ALL=("Crystalloid")
	#18	(ALL=(Colloid)) OR ALL=("Colloid" OR "Hydrocolloid")
	#19	ALL=(Plasma Volume)
	#20	(ALL=(Fluid Therapy)) OR ALL=("Therap*, Fluid" OR "Rehydrat*")
	#21	(ALL=(Resuscitation)) OR ALL=("restor*" OR "resuscitat*" OR "rehydrat*" OR "substitut*" OR "replac*")

	#22	#8 OR #9 OR #10 OR #11 OR #12 OR #13 OR #14 OR #15 OR #16 OR #17 OR #18 OR #19 OR #20 OR #21
	#23	#7 AND #22
	#24	ALL=(human*)
	#25	#23 AND #24
	#26	ALL=("random*" OR "RCT" OR "trial*" OR "randomized controlled trial*" OR "clin* trial")
	#27	#25 AND #27
**Cochrane**	#1	MeSH descriptor: [Shock, Hemorrhagic] explode all trees
	#2	MeSH descriptor: [Shock] explode all trees
	#3	("circulatory failure" OR "circulatory collapse" OR "hypovolemic shock"):ti,ab,kw
	#4	#2 OR #3
	#5	MeSH descriptor: [Wounds and Injuries] explode all trees
	#6	("trauma" OR "injury" OR "wound"):ti,ab,kw
	#7	#5 OR #6
	#8	#4 AND #7
	#9	#1 OR #8
	#10	MeSH descriptor: [Ringer's Lactate] explode all trees
	#11	Hartmanns Solution OR "Lactate Ringer"
	#12	MeSH descriptor: [Saline Solution] explode all trees
	#13	MeSH descriptor: [Albumins] explode all trees
	#14	Hydroxyethylated Starches OR "2-Hydroxyethyl Starches" OR "Hemohes" OR "Elohes" OR "Hespan" OR "Hetastarch" OR "Pentafraction" OR "Pentaspan" OR "Pentastarch" OR "Plasmasteril" OR "HAES-steril"
	#15	MeSH descriptor: [Dextrans] explode all trees
	#16	Dextran OR "Hemodex" OR "Hyskon" OR "Infukoll" OR "macrodex" OR "polyglucin" OR "Promit" OR "Rheoisodex" OR "Rondex" OR "Saviosol"
	#17	MeSH descriptor: [Gelatin] explode all trees

	#18	MeSH descriptor: [Crystalloid Solutions] explode all trees
	#19	Crystalloid
	#20	MeSH descriptor: [Colloids] explode all trees
	#21	Colloid OR "Hydrocolloid"
	#22	MeSH descriptor: [Plasma Volume] explode all trees
	#23	MeSH descriptor: [Fluid Therapy] explode all trees
	#24	Therapy, Fluid OR "Rehydration"
	#25	MeSH descriptor: [Resuscitation] explode all trees
	#26	restoration OR "resuscitation" OR "rehydration" OR "substitution" OR "replacing"
	#27	#10 OR #11 OR #12 OR #13 OR #14 OR #15 OR #16 OR #17 OR #18 OR #19 OR #20 OR #21 OR #22 OR #23 OR #24 OR #25 OR #26
	#28	#9 and #27
	#29	(Humans):ti,ab,kw
	#30	#28 AND #29
	#31	random OR "RCT" OR "trial" OR "randomized controlled trial" OR "clinical trial"
	#32	#30 AND #31
**Scopus**	#1	TITLE-ABS-KEY(Shock, Hemorrhagic)
	#2	TITLE-ABS-KEY("shock" OR "circulatory failure" OR "circulatory collapse" OR "hypovolemic shock")
	#3	TITLE-ABS-KEY("trauma*" OR "injur*" OR "wound*")
	#4	#2 AND #3
	#5	#1 AND #4
	#6	TITLE-ABS-KEY("Ringer's Lactate" OR "Hartmanns Solution" OR "Lactate* Ringer*")
	#7	TITLE-ABS-KEY(Ringer's Acetate)
	#8	TITLE-ABS-KEY(Saline Solution)
	#9	TITLE-ABS-KEY(Albumins)
	#10	TITLE-ABS-KEY("Hydroxyethyl Starch" OR "Hydroxyethylated Starches" OR "2-Hydroxyethyl Starches" OR "Hemohes" OR "Elohes"

		OR "Hespan" OR "Hetastarch" OR "Pentafraction" OR "Pentaspan" OR "Pentastarch" OR "Plasmasteril" OR "HAES-steril")
	#11	TITLE-ABS-KEY("Dextrans" OR "Dextran" OR "Hemodex" OR "Hyskon" OR "Infukoll" OR "*acrodex" OR "*olyglucin" OR "Promit" OR "Rheoisodex" OR "Rondex" OR "Saviosol")
	#12	TITLE-ABS-KEY(Gelatin)
	#13	TITLE-ABS-KEY("Crystalloid Solutions")
	#14	TITLE-ABS-KEY("Colloid" OR "Hydrocolloid")
	#15	TITLE-ABS-KEY(Plasma Volume)
	#16	TITLE-ABS-KEY("Fluid Therapy" OR "Therap*, Fluid" OR "Rehydrat*")
	#17	TITLE-ABS-KEY("Resuscitation" OR "restor*" OR "resuscitat*" OR "rehydrat*" OR "substitut*" OR "replac*")
	#18	#6 OR #7 OR #8 OR #9 OR #10 OR #11 OR #12 OR #13 OR #14 OR #15 OR #16 OR #17
	#19	#5 AND #18
	#20	TITLE-ABS-KEY(human)
	#21	#19 AND #20
	#22	TITLE-ABS-KEY("random*" OR "RCT" OR "trial*" OR "randomized controlled trial*" OR "clin* trial")
	#23	#21 AND #22
**CINAHL via EBSCO**	#1	MH Shock, Hemorrhagic
	#2	TX ("shock" OR "circulatory failure" OR "circulatory collapse" OR "hypovolemic shock")
	#3	TX ("trauma*" OR "injur*" OR "wound*")
	#4	S2 AND S3
	#5	S1 AND S4
	#6	MH ringer's lactate OR TX ("Hartmanns Solution" OR "Lactate* Ringer*")
	#7	MH Saline Solution
	#8	MH albumin

	#9	MH Hydroxyethyl Starch OR TX ("Hydroxyethylated Starches" OR "2-Hydroxyethyl Starches" OR "Hemohes" OR "Elohes" OR "Hespan" OR "Hetastarch" OR "Pentafraction" OR "Pentaspan" OR "Pentastarch" OR "Plasmasteril" OR "HAES-steril")
	#10	MH Dextrans OR TX ("Dextran" OR "Hemodex" OR "Hyskon" OR "Infukoll" OR "*acrodex" OR "*olyglucin" OR "Promit" OR "Rheoisodex" OR "Rondex" OR "Saviosol")
	#11	MH gelatin
	#12	MH crystalloid solutions
	#13	MH plasma volume
	#14	MH fluid therapy OR TX ("Therap*, Fluid" OR "Rehydrat*")
	#15	MH colloid OR TX hydrocolloid
	#16	MH resuscitation OR TX ("restor*" OR "resuscitat*" OR "rehydrat*" OR "substitut*" OR "replac*")
	#17	S6 OR S7 OR S8 OR S9 OR S10 OR S11 OR S12 OR S13 OR S14 OR S15 OR S16
	#18	S5 AND S17
	#19	TX (human)
	#20	S18 AND S19
	#21	TX "random*" OR "RCT" OR "trial*" OR "randomized controlled trial*" OR "clin* trial"
	#22	S20 AND S21
**PubMed**	#1	Shock, Hemorrhagic[MeSH Terms]
	#2	shock[MeSH Terms]
	#3	((circulatory failure[Title/Abstract]) OR (circulatory collapse[Title/Abstract])) OR (hypovolemic shock[Title/Abstract])
	#4	#2 OR #3
	#5	((trauma[MeSH Terms]) OR (injury[MeSH Terms])) OR (wound[MeSH Terms])
	#6	((trauma*[Title/Abstract]) OR (injur*[Title/Abstract])) OR (wound*[Title/Abstract])

#7 #5 OR #6

#8 #4 AND #7

#9 #1 OR #8

#10 Ringer's Lactate[MeSH Terms]

#11 "Hartmanns Solution"[Title/Abstract] OR "Lactate* Ringer*"[Title/Abstract]

#12 Ringer's Acetate[MeSH Terms]

#13 Saline Solution[MeSH Terms]

#14 Albumins[MeSH Terms]

#15 Hydroxyethyl Starch[MeSH Terms]

#16 "Hydroxyethylated Starches"[Title/Abstract] OR "Hemohes"[Title/Abstract] OR "Elohes"[Title/Abstract] OR "Hespan"[Title/Abstract] OR "Hetastarch"[Title/Abstract] OR "Pentafraction"[Title/Abstract] OR "Pentaspan"[Title/Abstract] OR "Pentastarch"[Title/Abstract] OR "Plasmasteril"[Title/Abstract] OR "HAES-steril"[Title/Abstract]

#17 Dextrans[MeSH Terms]

#18 "Dextran"[Title/Abstract] OR "Hemodex"[Title/Abstract] OR "Hyskon"[Title/Abstract] OR "Infukoll"[Title/Abstract] OR "macrodex"[Title/Abstract] OR "polyglucin"[Title/Abstract] OR "Promit"[Title/Abstract] OR "Rondex"[Title/Abstract] OR "Saviosol"[Title/Abstract]

#19 Gelatin[MeSH Terms]

#20 Crystalloid Solutions[MeSH Terms]

#21 Colloid[MeSH Terms]

#22 "Hydrocolloid"[Title/Abstract]

#23 Plasma Volume[MeSH Terms]

#24 Fluid Therapy[MeSH Terms]

#25 "Therapy, Fluid"[Title/Abstract] OR "Rehydrat*"[Title/Abstract]

#26 Resuscitation[MeSH Terms]

	#27	"restor*"[Title/Abstract] OR "resuscitat*"[Title/Abstract] OR "rehydrat*"[Title/Abstract] OR "substitut*"[Title/Abstract] OR "replac*"[Title/Abstract]
	#28	#10 OR #11 OR #12 OR #13 OR #14 OR #15 OR #16 OR #17 OR #18 OR #19 OR #20 OR #21 OR #22 OR #23 OR #24 OR #25 OR #26 OR #27
	#29	#9 AND #28
	#30	Humans[MeSH Terms]
	#31	#29 AND #30
	#32	"random*" OR "RCT" OR "trial*" OR "randomized controlled trial*" OR "clinical trial"
	#33	#31 AND #32
**ProQuest**	S1	mesh.Exact("Shock, Hemorrhagic")
	S2	mesh.Exact("Shock")
	S3	"circulatory failure" OR "circulatory collapse" OR "hypovolemic shock"
	S4	[S2] OR [S3]
	S5	mesh.Exact("wound and injuries")
	S6	"trauma" OR "injury" OR "wound"
	S7	[S5] OR [S6]
	S8	[S4] AND [S7]
	S9	[S1] AND [S8]
	S10	mesh.Exact("Ringer's Lactate")
	S11	"Hartmanns Solution" OR "Lactate* Ringer*"
	S12	mesh.Exact("Ringer's Acetate")
	S13	mesh.Exact("Saline Solution")
	S14	mesh.Exact("Albumins")
	S15	mesh.Exact("Hydroxyethyl Starch")
	S16	"Hydroxyethylated Starches" OR "2-Hydroxyethyl Starches" OR "Hemohes" OR "Elohes" OR "Hespan" OR "Hetastarch" OR "Pentafraction" OR "Pentaspan" OR "Pentastarch" OR "Plasmasteril" OR "HAES-steril"

S17	mesh.Exact("Dextrans")
S18	"Dextran" OR "Hemodex" OR "Hyskon" OR "Infukoll" OR "macrodex" OR "polyglucin" OR "Promit" OR "Rheoisodex" OR "Rondex" OR "Saviosol"
S19	mesh.Exact("Gelatin")
S20	mesh.Exact("Crystalloid Solutions")
S21	mesh.Exact("Colloid")
S22	"Hydrocolloid"
S23	mesh.Exact("Plasma Volume")
S24	mesh.Exact("Fluid Therapy")
S25	"Therap*, Fluid" OR "Rehydrat*"
S26	mesh.Exact("Resuscitation")
S27	"restor*" OR "resuscitat*" OR "rehydrat*" OR "substitut*" OR "replac*"
S28	[S10] OR [S11] OR [S12] OR [S13] OR [S14] OR [S15] OR [S16] OR [S17] OR [S18] OR [S19] OR [S20] OR [S21] OR [S22] OR [S23] OR [S24] OR [S25] OR [S26] OR [S27] OR [S28]
S29	[S9] AND [S28]
S30	mesh.Exact("humans")
S31	[S29] AND [S30]
S32	"random*" OR "RCT" OR "trial*" OR "randomized controlled trial*" OR "clin* trial"
S33	[S31] AND [S33]

Abbreviations: CINAHL, Cumulative Index to Nursing and Allied Health Literature

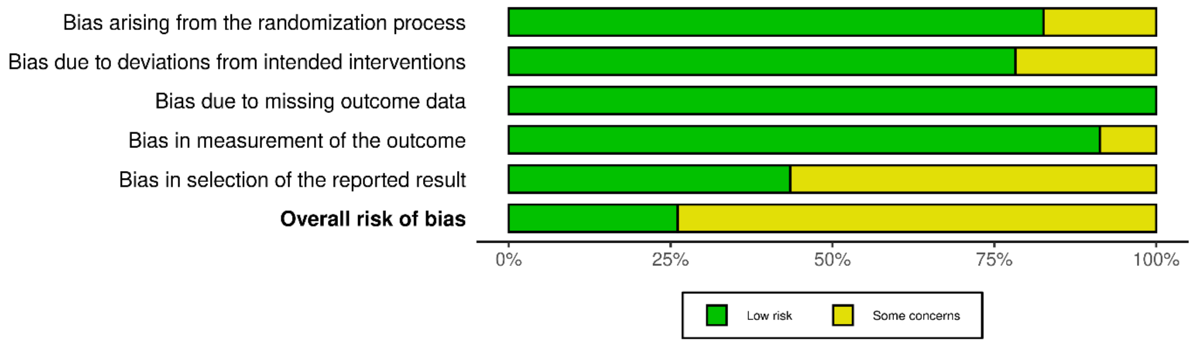

Figure S1. Domain-Specific Results of Quality Assessment of Included Studies Using the Risk of Bias 2 tool

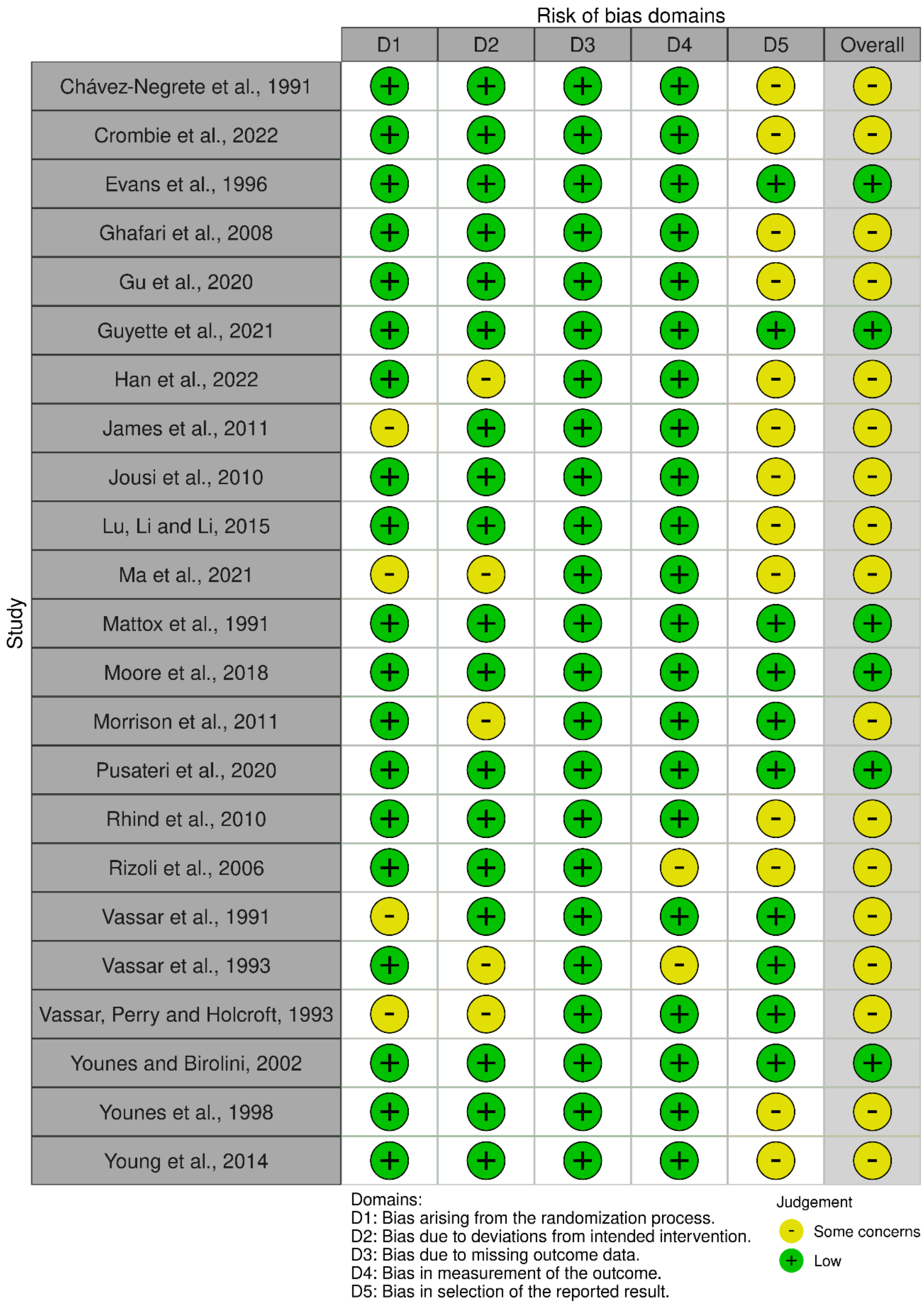

Figure S2. Detailed Quality Assessment Summary of Included Studies Using the Risk of Bias 2 tool

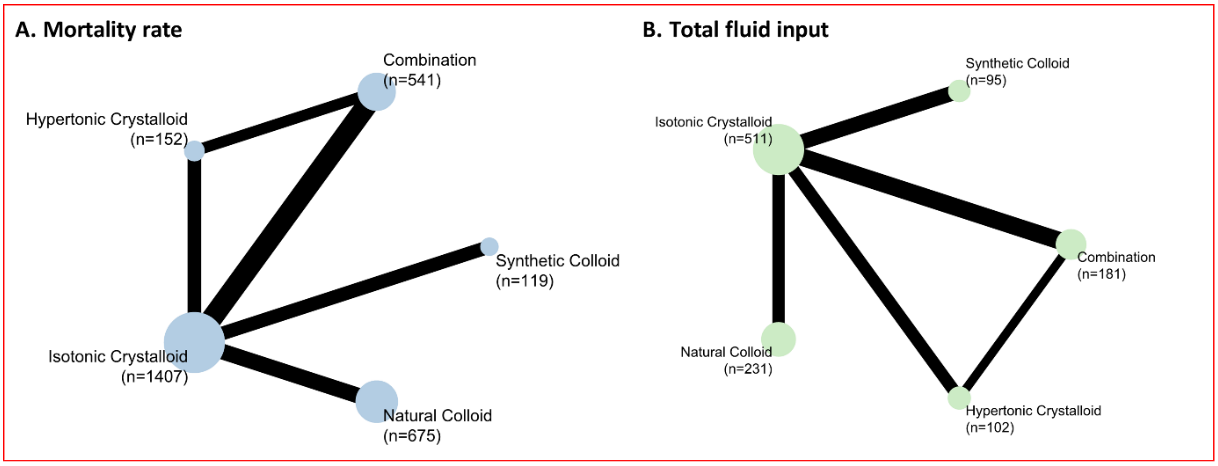

Figure S3. Network of Eligible Comparisons for Fluid Resuscitation in (A) Mortality Rate and (B) Total Fluid Input Outcomes

The width of the line indicates the number of direct pairwise comparisons. The width of the node indicates the number of samples contained in the treatment.

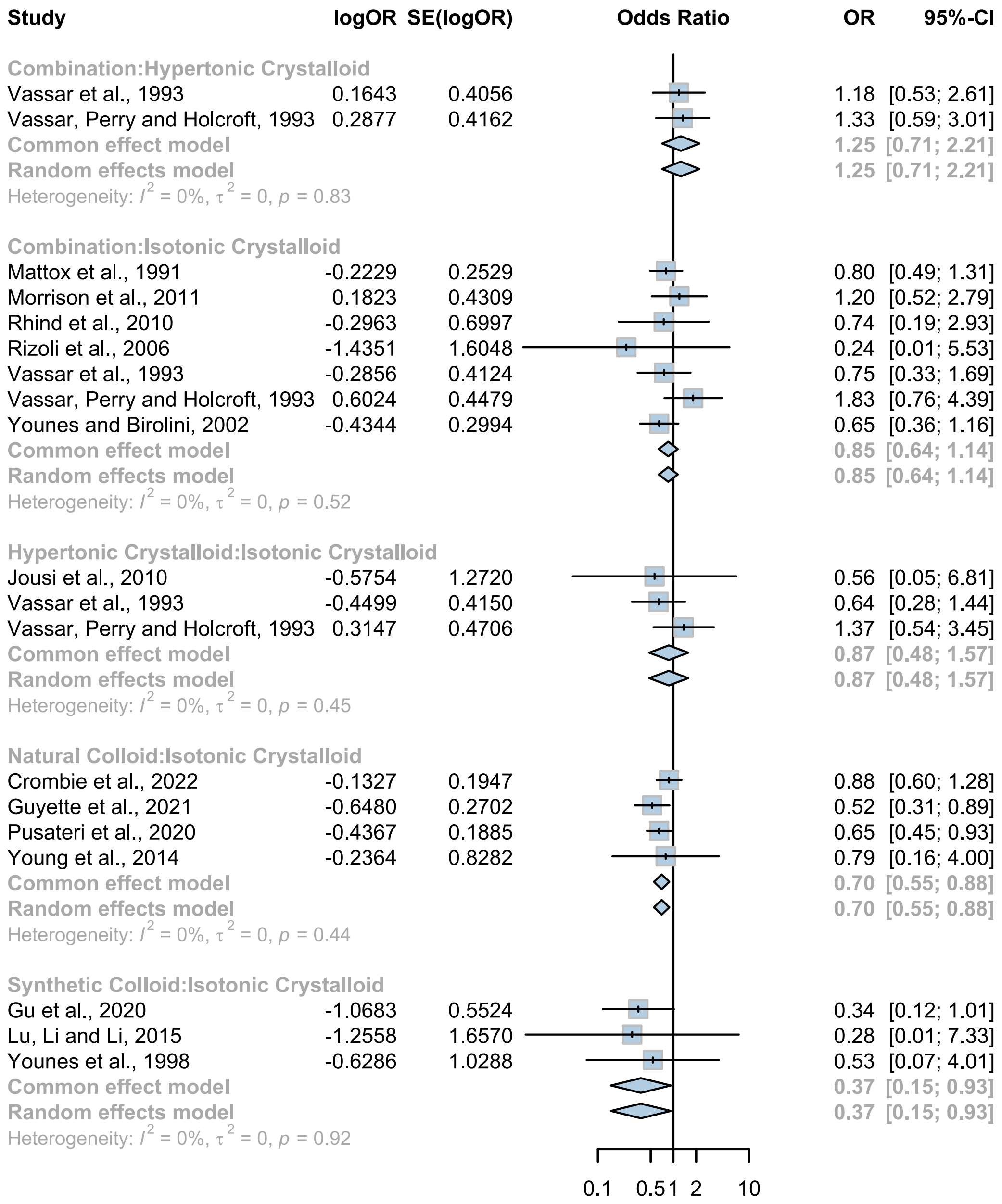

Figure S4. Pairwise Forest Plot of Individual Studies in Mortality Rate Network Meta-Analysis

The width of the line indicates the number of direct pairwise comparisons. The width of the node indicates the number of samples contained in the treatment. Estimates to the left of the line favor the compared interventions, while estimates to the right favor the reference interventions.

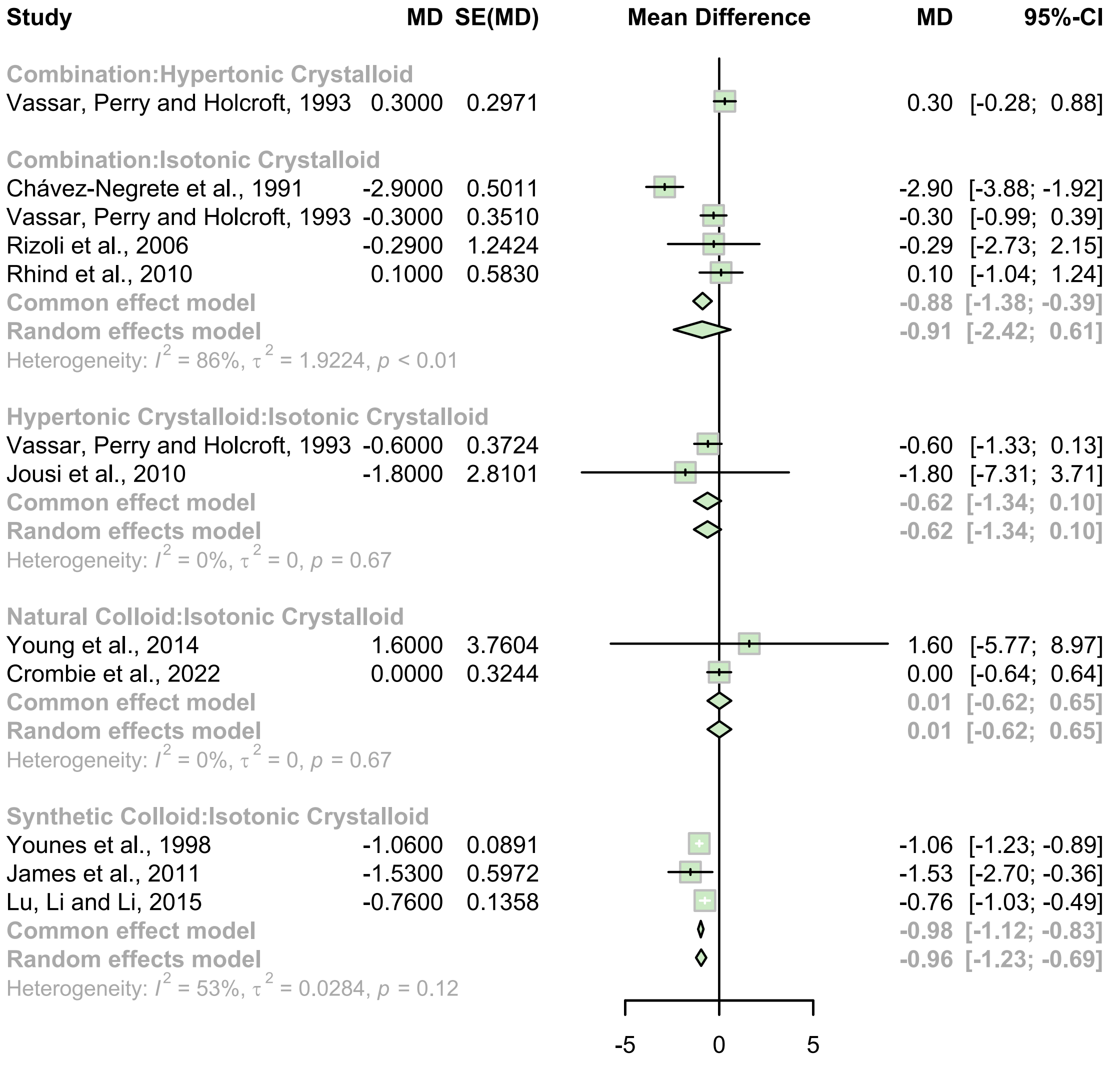

Figure S5. Pairwise Forest Plot of Individual Studies in Total Fluid Input Network Meta-Analysis

The width of the line indicates the number of direct pairwise comparisons. The width of the node indicates the number of samples contained in the treatment. Estimates to the left of the line favor the compared interventions, while estimates to the right favor the reference interventions.

Table S3. League Table of Frequentist Random-Effects Model Network Meta-Analysis

A. Mortality Rate

**Combination**	1.25 (0.71; 2.21)	0.85 (0.64; 1.14)	N.A	N.A
1.14 (0.68; 1.91)	**Hypertonic Crystalloid**	0.87 (0.48; 1.57)	N.A	N.A
0.85 (0.64; 1.14)	0.75 (0.44; 1.26)	**Isotonic Crystalloid**	1.43 (1.13; 1.81)	2.70 (1.08; 6.74)
1.22 (0.84; 1.77)	1.07 (0.60; 1.90)	1.43 (1.13; 1.81)	**Natural Colloid**	N.A
2.29 (0.88; 5.99)	2.02 (0.70; 5.78)	2.70 (1.08; 6.74)	1.88 (0.73; 4.85)	**Synthetic Colloid**

B. Total Fluid Input

**Combination**	0.30 (-0.78; 1.38)	-0.95 (-1.67; -0.23)	N.A	N.A
0.04 (-0.96; 1.05)	**Hypertonic Crystalloid**	-0.65 (-1.79; 0.49)	N.A	N.A
-0.96 (-1.67; -0.24)	-1.00 (-2.03; 0.03)	**Isotonic Crystalloid**	-0.04 (-1.13; 1.06)	1.02 (0.41; 1.62)
-0.99 (-2.30; 0.32)	-1.04 (-2.54; 0.47)	-0.04 (-1.13; 1.06)	**Natural Colloid**	N.A
0.06 (-0.88; 1.00)	0.02 (-1.18; 1.21)	1.02 (0.41; 1.62)	1.05 (-0.20; 2.31)	**Synthetic Colloid**

Values are presented as odds ratio (OR) for mortality rate, and mean difference (MD) for total fluid input.

Table S4. Heterogeneity, Local, and Global Inconsistency Assessment for the Network Meta-Analysis

A. Mortality Rate

Arm 1	Arm 2	k	I^2	Direct estimate	Indirect estimate	Network meta-analysis	Local Inconsistency	Global Inconsistency
Combination	Hypertonic Crystalloid	2	0.00%	1.25 [0.71; 2.21]	0.74 [0.22; 2.49]	1.14 [0.68; 1.91]	0.4451	Q = 5.83 p = 0.054
Combination	Isotonic Crystalloid	7	0.00%	0.85 [0.64; 1.14]	0.38 [0.01; 23.47]	0.85 [0.64; 1.14]	0.7031	
Combination	Natural Colloid	0	N.A	N.A	1.22 [0.84; 1.77]	1.22 [0.84; 1.77]	N.A	
Combination	Synthetic Colloid	0	N.A	N.A	2.29 [0.88; 5.99]	2.29 [0.88; 5.99]	N.A	
Hypertonic Crystalloid	Isotonic Crystalloid	3	0.00%	0.87 [0.48; 1.57]	0.44 [0.15; 1.34]	0.75 [0.44; 1.26]	0.2918	
Hypertonic Crystalloid	Natural Colloid	0	N.A	N.A	1.07 [0.60; 1.90]	1.07 [0.60; 1.90]	N.A	
Hypertonic Crystalloid	Synthetic Colloid	0	N.A	N.A	2.02 [0.70; 5.78]	2.02 [0.70; 5.78]	N.A	
Natural Colloid	Isotonic Crystalloid	4	0.00%	0.70 [0.55; 0.88]	N.A	0.70 [0.55; 0.88]	N.A	

Synthetic Colloid	Isotonic Crystalloid	3	0.00%	0.37 [0.15; 0.93]	N.A	0.37 [0.15; 0.93]	N.A	
Natural Colloid	Synthetic Colloid	0	N.A	N.A	1.88 [0.73; 4.85]	1.88 [0.73; 4.85]	N.A	

B. Total Fluid Input

Arm 1	Arm 2	k	I^2	Direct estimate	Indirect estimate	Network meta-analysis	Local Inconsistency	Global Inconsistency
Combination	Hypertonic Crystalloid	1	N.A	0.30 [-0.78; 1.38]	-1.53 [-4.22; 1.15]	0.04 [-0.96; 1.05]	0.2147	
Combination	Isotonic Crystalloid	4	86.40%	-0.95 [-1.67; -0.23]	-2.39 [-12.26; 7.48]	-0.96 [-1.67; -0.24]	0.7753	
Combination	Natural Colloid	0	N.A	N.A	-0.99 [-2.30; 0.32]	-0.99 [-2.30; 0.32]	N.A	
Combination	Synthetic Colloid	0	N.A	N.A	0.06 [-0.88; 1.00]	0.06 [-0.88; 1.00]	N.A	Q = 1.12 p = 0.571
Hypertonic Crystalloid	Isotonic Crystalloid	2	0.00%	-0.65 [-1.79; 0.49]	-2.56 [-4.96; -0.15]	-1.00 [-2.03; 0.03]	0.161	
Hypertonic Crystalloid	Natural Colloid	0	N.A	N.A	-1.04 [-2.54; 0.47]	-1.04 [-2.54; 0.47]	N.A	
Hypertonic Crystalloid	Synthetic Colloid	0	N.A	N.A	0.02 [-1.18; 1.21]	0.02 [-1.18; 1.21]	N.A	

Natural Colloid	Isotonic Crystalloid	2	0.00%	0.04 [-1.06; 1.13]	N.A	0.04 [-1.06; 1.13]	N.A
Synthetic Colloid	Isotonic Crystalloid	3	53.30%	-1.02 [-1.62; -0.41]	N.A	-1.02 [-1.62; -0.41]	N.A
Natural Colloid	Synthetic Colloid	0	N.A	N.A	1.05 [-0.20; 2.31]	1.05 [-0.20; 2.31]	N.A

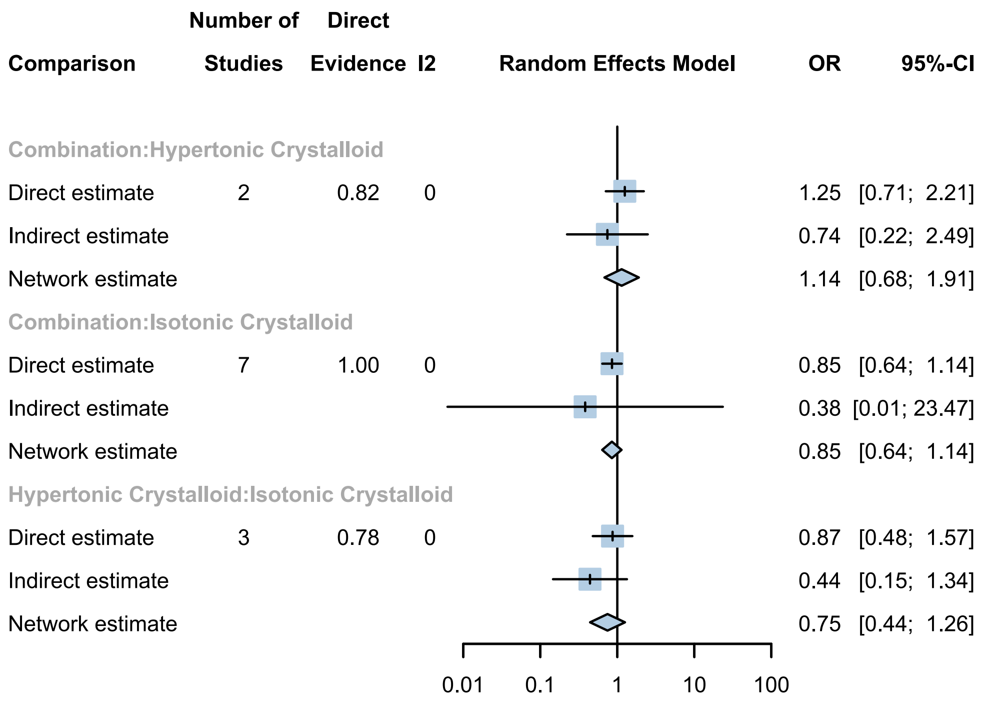

Figure S6. Netsplit Forest Plot of Local Inconsistency Assessment for Mortality Rate Network Meta-Analysis

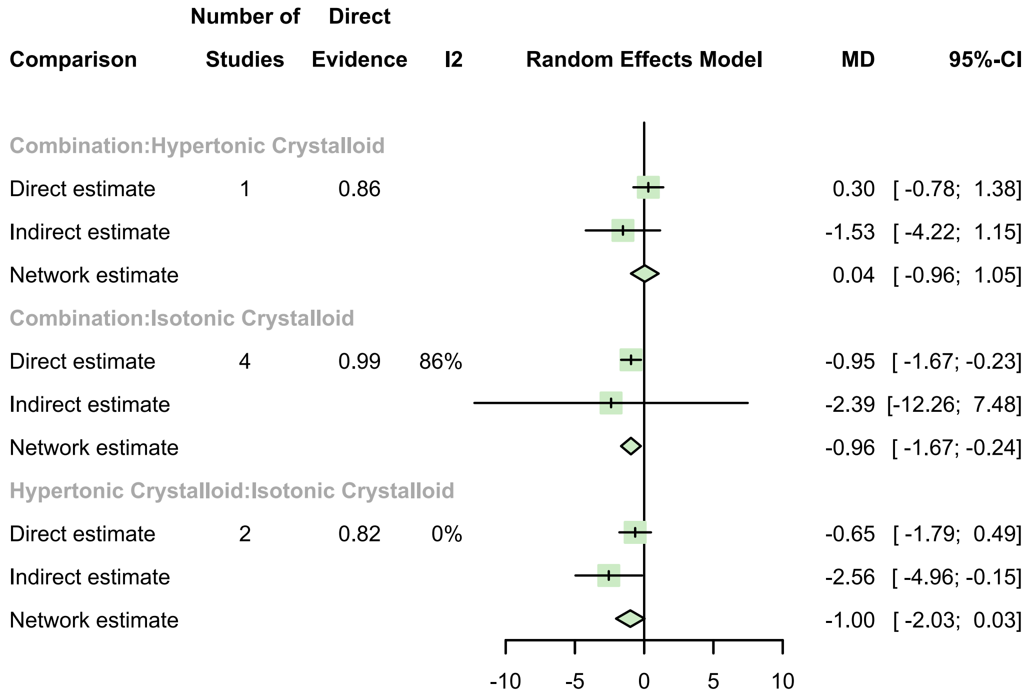

Figure S7. Netsplit Forest Plot of Local Inconsistency Assessment for Total Fluid Input Network Meta-Analysis

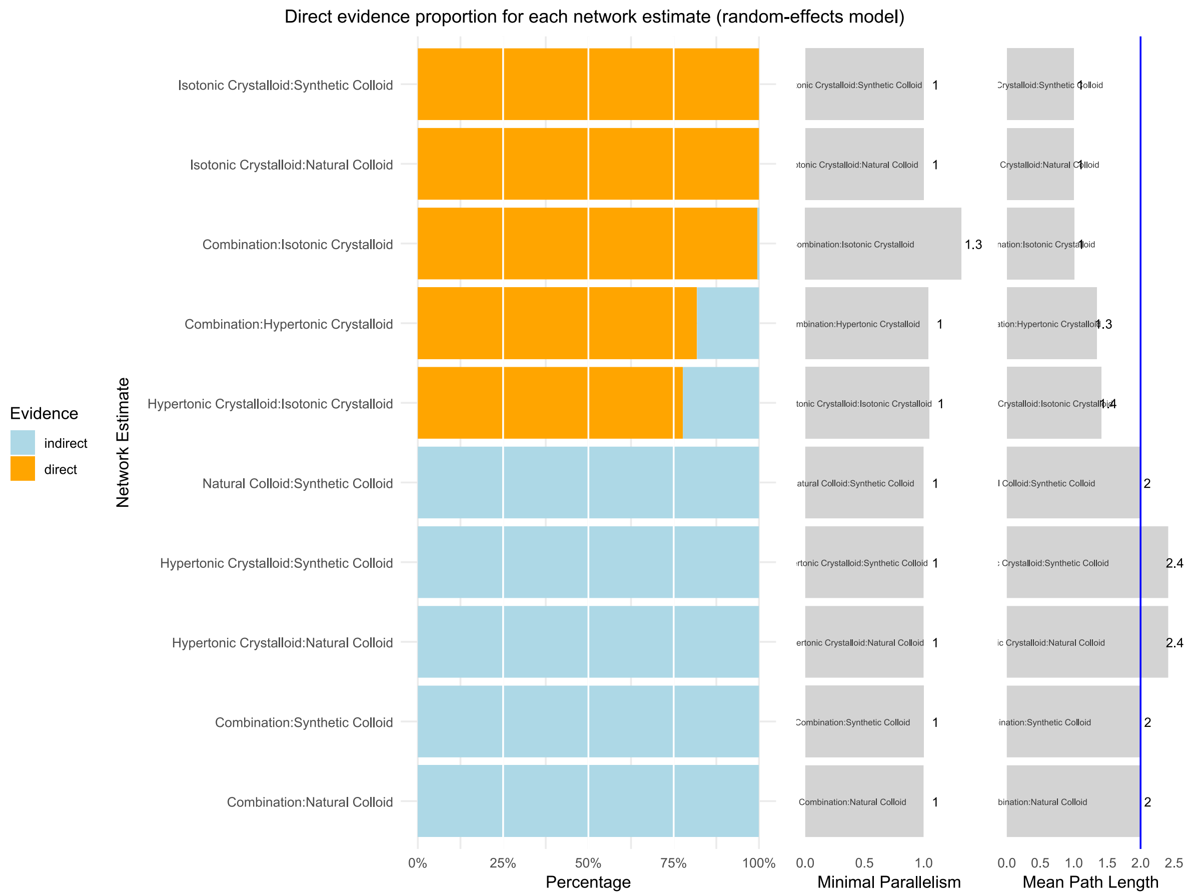

Figure S8. Direct and Indirect Evidence Plot of Mortality Rate Network Meta-Analysis

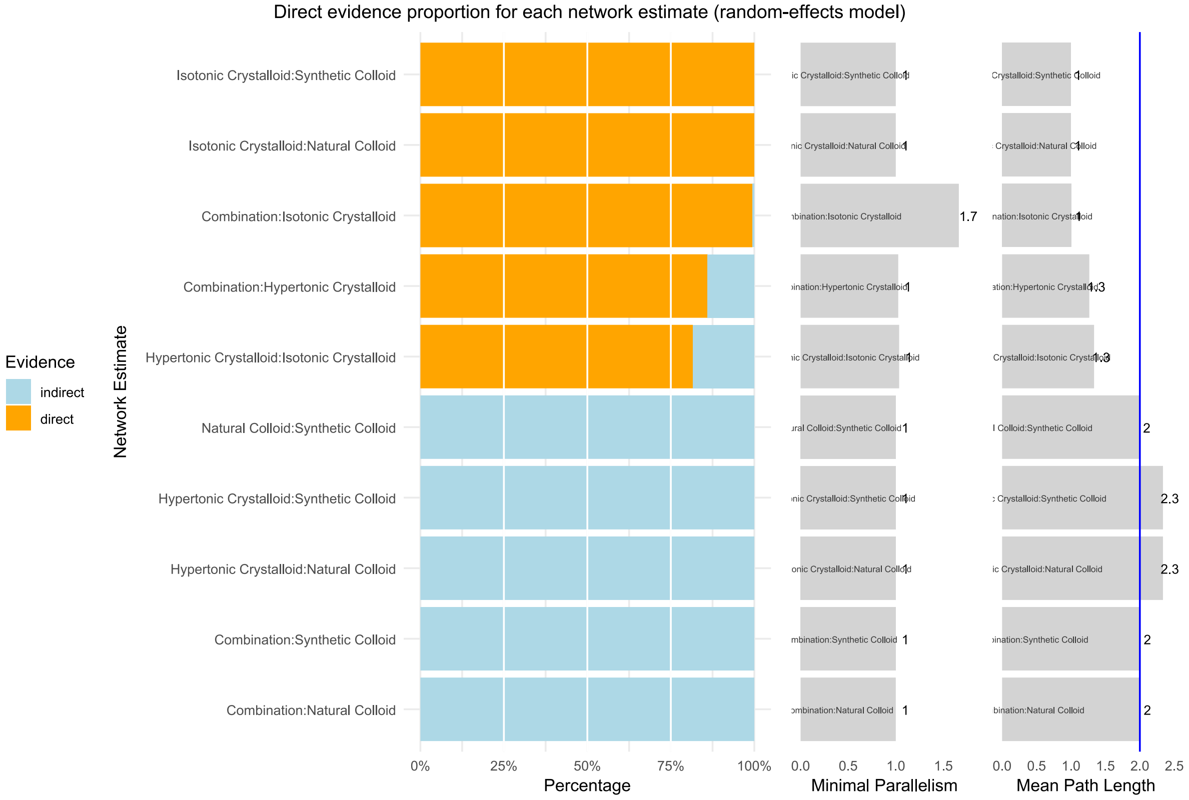

Figure S9. Direct and Indirect Evidence Plot of Total Fluid Input Network Meta-Analysis

Table S5. Summary of Subgroup and Network Meta-Regression Analyses from Network Meta-Analyses

Subgroup Analyses

Outcome measure	Variable	Subgroup	B	L CrI	U CrI	Significancy
Mortality	Continent	America<br>Non-America	-0.275	-0.863	0.381	Not significant
	Characteristics	Hemorrhagic shock<br>Trauma hemorrhagic shock	-0.374	-1.121	0.341	Not significant
	Analysis	ITT<br>PP	-0.082	-1.539	1.413	Not significant
	Risk of bias	Low risk<br>Moderate and high risk	0.348	-0.097	0.778	Not significant
Fluid input	Continent	America<br>Non-America	0.382	-1.487	2.854	Not significant
	Characteristics	Hemorrhagic shock<br>Trauma hemorrhagic shock	-0.881	-3.662	1.079	Not significant
	Analysis	ITT<br>PP	-0.844	-6.301	4.017	Not significant
	Risk of bias	Low risk<br>Moderate and high risk	-1.024	-15.501	5.021	Not significant

Network Meta-Regression Analyses

Outcome measure	Covariate	B	L CrI	U CrI	Significancy
Mortality	Publication year	-0.162	-0.97	0.686	Not significant
	Mean age	-0.104	-0.554	0.315	Not significant
	Sample size	-0.002	-0.594	0.666	Not significant

	adherence rate	-0.009	-0.624	0.465	Not significant
	Female	-0.247	-1.354	0.707	Not significant
	GCS	0.067	-0.787	0.998	Not significant
	ISS	0.114	-0.464	0.598	Not significant
	time to emergency	-0.546	-2.025	0.661	Not significant
	SBP	-0.563	-1.826	0.675	Not significant
**Fluid Input**	Publication year	0.774	-2.061	3.191	Not significant
	Mean age	0.243	-2.355	2.757	Not significant
	Sample size	0.454	-2.634	4.443	Not significant
	adherence rate	0.118	-2.078	2.007	Not significant
	Female	0.564	-4.522	2.717	Not significant
	GCS	-0.511	-4.279	2.677	Not significant
	ISS	0.288	-1.696	2.373	Not significant
	time to emergency	0.19	-2.653	3.165	Not significant
	pH	-1.02	2.043	-4.089	Not significant
	Hemoglobin	-0.169	-3.137	2.701	Not significant

lactate	0.09	-3.921	3.835	Not significant
SBP	0.587	-1.705	3.537	Not significant

B, Beta coefficient**; L CrI**, Lower 95% credible interval; **U CrI**, Upper 95% credible interval; **GCS**, Glasgow coma scale; **ISS**, injury severity score; **SBP**, systolic blood pressure; **ITT**, intention-to-treat; **PP**, per protocol.

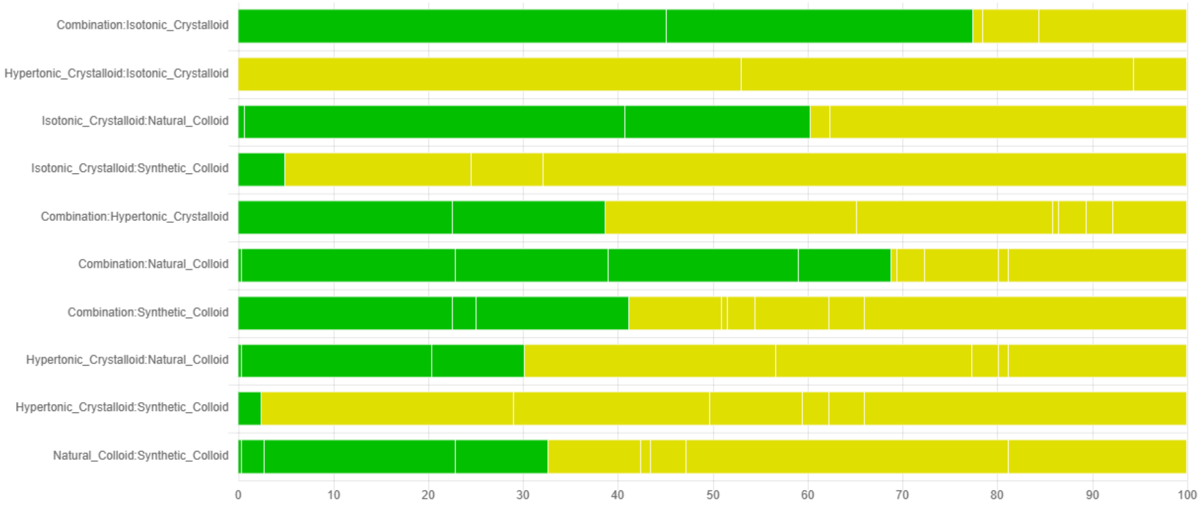

Figure S10. Risk of Bias Contributions Within-Study Bias for Network Meta-Analysis of Mortality Rate

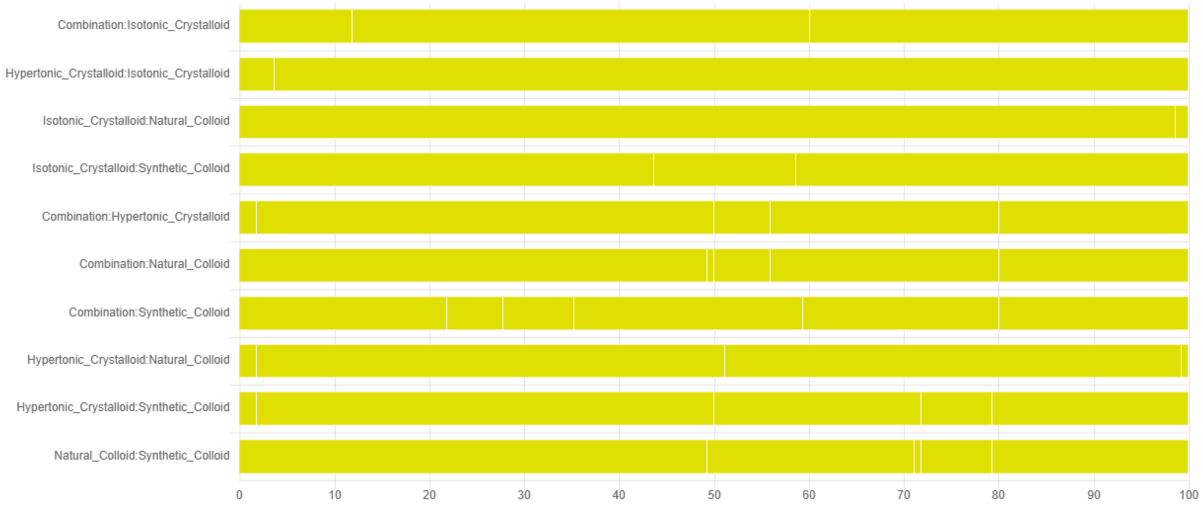

Figure S11. Risk of Bias Contributions Within-Study Bias for Network Meta-Analysis of Total Fluid Input

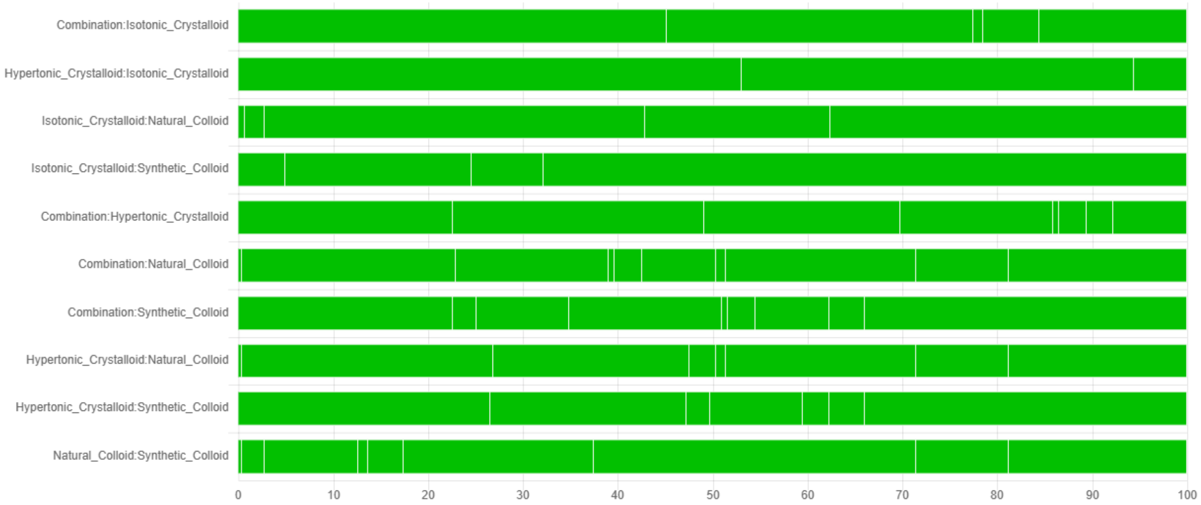

Figure S12. Indirectness Contributions in GRADE Reports for Network Meta-Analysis of Mortality Rate

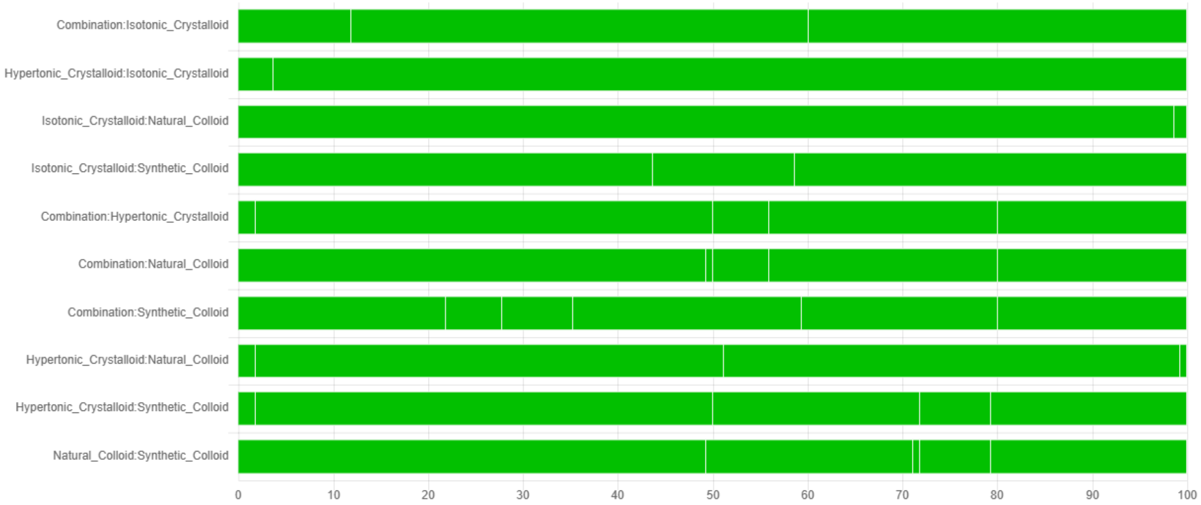

Figure S13. Indirectness Contributions in GRADE Reports for Network Meta-Analysis of Total Fluid Input
